# Phenylacetyl glutamine: a novel biomarker for stroke recurrence warning

**DOI:** 10.1186/s12883-023-03118-5

**Published:** 2023-02-16

**Authors:** Li Ma, Guoping Fu, Rongrong Liu, Feng Zhou, Shiye Dong, Yang Zhou, Jingwei Lou, Xinjun Wang

**Affiliations:** 1 Department of Neurology, Shaoxing Second Hospital, The Second Affiliated Hospital of Shaoxing University of Arts and Sciences, Shaoxing, 312000 China; 2 Shanghai Zhangjiang Institute of Medical Innovation, Shanghai, 201204 China; 3 Molecular Medicine Center, Shaoxing Second Hospital, The Second Affiliated Hospital of Shaoxing University of Arts and Sciences, Shaoxing, 312000 China; 4 Translational Medical Center for Stem Cell Therapy and Institute for Regenerative Medicine, Shanghai East Hospital, Shanghai Key Laboratory of Signaling and Disease Research, School of Life Sciences and Technology, Tongji University, Shanghai, 200092 China; 5 Shanghai Institution of Gut Microbiota Research and Engineering Development, Tenth People’s Hospital of Tongji University, Tongji University School of Medicine, Shanghai, 200072 China

**Keywords:** Phenylacetyl glutamine, Stroke, LASSO regression, Risk factors, Machine learning

## Abstract

**Background:**

Stroke is the second leading cause of disease-related death and the third leading cause of disability worldwide. However, how to accurately warn of stroke onset remains extremely challenging. Recently, phenylacetyl glutamine (PAGln) has been implicated in the onset of stroke, but evidences from cohort studies of onset are lacking, especially in patients with first-onset or recurrent. It is necessary to deeply demonstrate the effectiveness of PAGln level on warning stroke onset.

**Methods:**

One hundred fifteen first onset stroke patients, 33 recurrent stroke patients, and 135 non-stroke controls were included in the analysis. Risk factors associated with stroke attacking were evaluated, and plasma PAGln levels were detected via HPLC-MS based method. LASSO regression, Pearson correlation analysis, and univariate analysis were carried out to demonstrate the associations between PAGln levels and risk factors of stroke. Random forest machine learning algorithm was used to build classification models to achieve the distinction of first-onset stroke patients, recurrent stroke patients, and non-stroke controls, and further demonstrate the contribution of PAGln levels in the distinction of stroke onset.

**Results:**

The median level of PAGln in the first-onset stroke group, recurrent stroke group, and non-stroke group was 933 ng/mL, 1014 ng/mL, and 556 ng/mL, respectively. No statistical correlation was found between PAGln level and subject’s living habits, eating preferences, and concomitant diseases (hypertension, hyperlipidemia, and diabetes). Stroke severity indicators, mainly age and NIHSS score, were found associate with the PAGln levels. Machine learning classification models confirmed that PAGln levels, as the main contributing variable, could be used to distinguish recurrent stroke patients (but not first-onset stroke patients) from non-stroke controls.

**Conclusion:**

PAGln may be an effective indicator to monitor the recurrence in stroke patients.

**Supplementary Information:**

The online version contains supplementary material available at 10.1186/s12883-023-03118-5.

Stroke is a cerebrovascular disease with a high incidence, high fatality rate, and high disability rate [1]. It also is the world’s second leading cause of disease-related death and the third leading cause of disability in 2019 [2]. Over the past decade, the burden of stroke is increasing rapidly in low- and middle-income countries, and many of these countries cannot cope with the challenges stroke brings [2]. Rapid, sensitive, and accurate assessment of the stroke-onset-risk and timely treatment and/or rescue of patients are of great significance for reducing the adverse events caused by stroke.

For the Chinese Han population, ischemic stroke accounts for about 60–80% of all strokes [3]. Stenosis of the extracranial internal carotid artery is the main cause of ischemic stroke, which is estimated to cause 8 to 15% of ischemic stroke [4]. Clinical and imaging features, including intraplaque hemorrhage, impaired cerebrovascular reserve, carotid plaque echo, lipid-rich necrotic core, etc., have been determined to be related to carotid artery stenosis and the onset of stroke [5]. However, these pieces of evidences cannot fully explain the pathophysiological mechanism of stroke onset [6]. Therefore, identifying new risk factors is essential to improve the understanding of the causes and prognosis of ischemic stroke patients, which helps to accurately assess the risk of stroke and promptly intervene.

Recently, some of metabolites derived from gut microbes have been identified as contributing factors to cardiovascular and cerebrovascular diseases (CVD) [6, 7]. Among them, phenylacetylglutamine (PAGln) can be associated with CVD events through a variety of mechanisms. First, PAGln is involved in thrombus formation through complementary pathways, including enhanced platelet function and stimulus-dependent response to agonists or intracellular calcium release [8]. Arterial injury model studies have further confirmed that pathological levels of PAGln can promote the incidence of thrombus [8]. Moreover, PAGln can interact with G protein-coupled receptors (GPCRs), such as the adrenergic receptors (ADRs) (mainly α2A, α2B, and β2- ADRs), to mediate cell responses and downstream signal transduction [8]. These ADRs are widely expressed on cell membrane of platelets and can regulate myocardial and vascular functions. Although the pathogenic role of PAGln in cardiovascular and cerebrovascular diseases has received extensive attention in the past 2 years, there is less evidence for the relationship between PAGln levels at onset and stroke attacking, especially in Asian populations.

In this study, we focused on the application of PAGln monitoring in early warning of stroke onset. We recruited 283 participants, containing 115 first onset stroke patients, 33 recurrent stroke patients, and 135 non-stroke controls. LASSO regression, multivariate analysis, and univariate analysis were performed to demonstrate the association between PAGln levels and stroke risk factors. Random forest machine learning algorithm was used to build classification models to elucidate the contribution of PAGln level in distinguishing stroke patients from non-stroke controls.

## Methods and materials

### Study population

From September 2020 to September 2021, 283 stroke or suspected stroke patients came to the Shaoxing Second Hospital in China receiving emergency treatment. Based on results of diagnosis and medical history investigation, 115 were first onset stroke patients, 33 were recurrent stroke patients, and 135 were non-stroke patients. Subjects’ medical history, medication history, diet preference, living habits, and physical indexes were collected (Supplementary information 1). All subjects received an ultrasonic diagnosis to detect carotid intima-media thickness (IMT) and the size and number of carotid plaques. Stroke subjects were additionally evaluated by the NIH stroke scale (NIHSS) and modified Rankin scale (MRS). Peripheral blood samples of subject was collected within 6 hours after stroke rescue to detect PAGln and blood lipid levels.

### Definition of stroke patients and non-stroke controls

Stroke patients was acute onset with symptoms of focal neurological deficits, such as numbness, weakness, and language barriers on one side of the face or limbs. Responsible lesions appear on imaging, or stroke symptoms and signs persist for more than 24 hours. Patients with IMT ≤ 1.5 mm and no stroke lesions were considered non-stroke controls. Stroke-like diseases patients, such as multiple sclerosis, were excluded in our study. Patients who had taken antibiotics in the week before admission were also excluded.

### Sample collection and detection

Peripheral blood samples were collected using EDTA-K2 anticoagulant tubes. Invert the anticoagulation tube several times and centrifuge at 300 g to separate the plasma. Each plasma sample was divided into 3 aliquots. One aliquot of them was sent to the Clinical Laboratory for blood lipid testing. Another aliquot was used for PAGln detection. The rest of the aliquot was stored at − 80 °C for outlier verification. Permission for the use of blood samples was obtained from the Ethics Committee of the Shaoxing Second Hospital.

### PAGln detection

The LC-MS/MS-based stable isotope internal standard strategy is used to quantify the concentration of PAGln in plasma samples. Specifically, 650 μL of acetonitrile solution containing 80 ng/mL PAGln-d5 was added to 200 μL of plasma samples. After centrifugation (1000 g, 3 min), the supernatant was collected and evaporated under the N_2_ atmosphere. Then resuspended the residue in 200 μL deionized water, and took 160 μL for subsequent testing. LC-MS/MS analysis was performed using an ACQUITY UPLC I-Class/Xevo TQD system equipped with an ACQUITY UPLC HSS T3 column (2.1 × 100 mm; Waters, USA). The mobile phase consisted of (A) 0.1% formic acid aqueous solution and (B) pure methanol. The flow rate of the mobile phase was 0.3 mL/min. The elution procedure was performed according to the following gradient: 0 minutes, 2% B; 0–1.0 minutes, 2% B; 1.0–2.5 minutes, 50% B; 2.5–5.0 minutes, 2% B. A sample volume of 10 μL was used for injection. Use the following parameters to operate electrospray ionization for TMAO detection in positive ion mode: capillary voltage 3.5 kV; cone voltage 30 V; collision energy 18 V; desolventizing temperature 350 °C; desolventizing gas flow rate 650 L/h. The characteristic ion pairs for PAGln and PAGln-d5 detection were m/z 265.2 → 130.5 and m/z 270.0 → 130.2, respectively.

### LASSO regression analysis

LASSO regression analysis was performed to select variables that potentially contributed to TMAO levels. In order to determine the penalty factor, a 10-fold cross-validation test was carried out. According to the principle reported by Li et al. [9], the best lambda for variable filtering was obtained.

### Statistical analysis

SPSS 19.0 software was used for statistical analysis. Continuous variables were represented as the median (interquartile range). Discrete variables were considered categorical variables and expressed as the percentage of eligible subjects. Differences of continuous variables with normal distribution among groups were compared by the t-test, while nonparametric tests or ANOVA design with a post hoc test were used if they are not consistent with normal distribution. Differences of discrete variables were compared by the Chi-square test. Only *p*-value < 0.05 was considered statistically significant. Random forest machine learning was performed using ‘Wu Kong’ platform (https://www.omicsolution.com/wkomics/main/).

## Results

### Cohort characteristics

From September 2020 to September 2021, we rescued 283 patients with stroke or suspected stroke in the Shaoxing Second Hospital. Among them, 115 are first onset stroke patients, 33 are recurrent stroke patients, and 135 are diagnosed as non-stroke patients. The main baseline characteristics of the cohort are listed in Table 1 (and Table S1 in Supplementary information 1). Both first-onset stroke patients and recurrent stroke patients in our cohort are mainly ischemic. Nearly half of the subjects in stroke patients had taken aspirin within 1 week, compared with only 23.7% of subjects in the control group. Moreover, stroke subjects have a higher proportion of cardiovascular complications and unhealthy living habits, relative to non-stroke controls. But in terms of disease history, including hypertension, hyperlipidemia, and diabetes, there was no statistically significant difference between the three groups of subjects.

**Table 1:** Baseline characteristics of the cohort

Variables	First-Onset Stroke Patients (***N*** = 115)	Recurrent Stroke Patients (***N*** = 33)	Non-Stroke Controls (***N*** = 135)	***p***-value
PAGln, ng/mL	933 (510–1445)	1014 (490–1590)	556 (293–1023)	0.008
Age, yrs	70 (64–80)	71 (65–76)	67 (61–73)	0.008
Male, %	68.70	75.76	50.37	0.002
Ischemic, %	95.65	93.94	0.00	/
Hemorrhagic, %	0.00	3.03	0.00	/
Aspirin usage, %	44.35	57.58	23.70	< 0.001
Diabetes history, %	28.15	39.39	27.83	0.401
Hypertension history, %	66.96	81.82	61.48	0.084
Hyperlipidemia history, %	4.35	15.15	11.85	0.055
Cardiovascular complications, %	21.74	30.30	12.59	0.030
Former smokers, %	9.57	18.18	6.67	0.120
Smokers, %	23.48	21.21	14.07	0.153
Non-smokers, %	66.96	60.61	79.26	0.028
Former drinkers, %	4.35	18.18	4.44	0.007
Drinkers, %	30.43	15.15	17.78	0.032
Non-drinkers, %	65.22	66.67	77.78	0.074
FBG, mM	5.43 (4.85–7.37)	5.69 (4.94–6.71)	5.23 (4.62–6.67)	0.095
DBP, mmHg	88 (77–96)	86 (76–94)	82 (75–90)	0.011
SBP, mmHg	154 (140–173)	159 (147–168)	143 (130–155)	< 0.001
Homocysteine, M	13.55 (10.53–17.60)	12.90 (10.40–16.30)	11.00 (9.50–13.10)	0.31
TC, mM	4.54 (4.00–5.17)	3.81 (3.24–4.45)	4.44 (3.70–5.13)	0.004
TG, mM	1.19 (0.91–1.56)	0.94 (0.73–1.31)	1.26 (0.88–1.83)	0.147
HDL-C, mM	1.15 (0.96–1.30)	0.94 (0.83–1.20)	1.15 (0.97–1.39)	0.011
LDL-C, mM	3.06 (2.57–3.44)	2.61 (1.95–2.93)	2.90 (2.34–3.48)	0.014
Lipoprotein A, mM	148.50 (69.00–257.25)	124.00 (65.00–227.00)	141.00 (71.00–216)	0.616
Apolipoprotein A1, g/L	1.18 (1.05–1.28)	1.07 (0.93–1.23)	1.23 (1.08–1.38)	< 0.001
Apolipoprotein B, g/L	0.89 (0.74–0.98)	0.78 (0.62–0.87)	0.84 (0.68–1.03)	0.542
LDL-1, mg/dL	29.00 (19.50–38.00)	26.00 (18.00–33.00)	26.00 (17.50–35.00)	0.411
LDL-2, mg/dL	23.00 (18.00–28.00)	22.00 (16.00–29.00)	21.00 (14.00–27.00)	0.184
LDL-3, mg/dL	10.00 (4.00–17.00)	7.00 (4.00–14.00)	9.00 (4.00–16.00)	0.569
LDL-4, mg/dL	3.00 (0.00–7.00)	2.00 (0.00–4.00)	2.00 (0.00–7.50)	0.376
LDL-5, mg/dL	0.00 (0.00–1.00)	0.00 (0.00–0.00)	0.00 (0.00–1.00)	0.41
LDL-6, mg/dL	0.00 (0.00–0.00)	0.00 (0.00–0.00)	0.00 (0.00–0.00)	0.879
LDL-7, mg/dL	0.00 (0.00–0.00)	0.00 (0.00–0.00)	0.00 (0.00–0.00)	0.969

Values are median (interquartile range) or percentage. The *p*-value for continuous variables among three groups was obtained by ANOVA design (with a post hoc test). The *p*-value for discrete variables among three groups was obtained by chi-square test

*PAGln* phenylacetyl glutamine, *FBG* fasting blood-glucose, *DBP* diastolic blood pressure, *SBP* systolic blood pressure, *TC* total cholesterol, *TG* triacylglycerol, *TCHO* total cholesterol, *HDL-C* high-density lipoprotein-cholesterol, *LDL* low-density lipoprotein- cholesterol

**Fig. 1:**
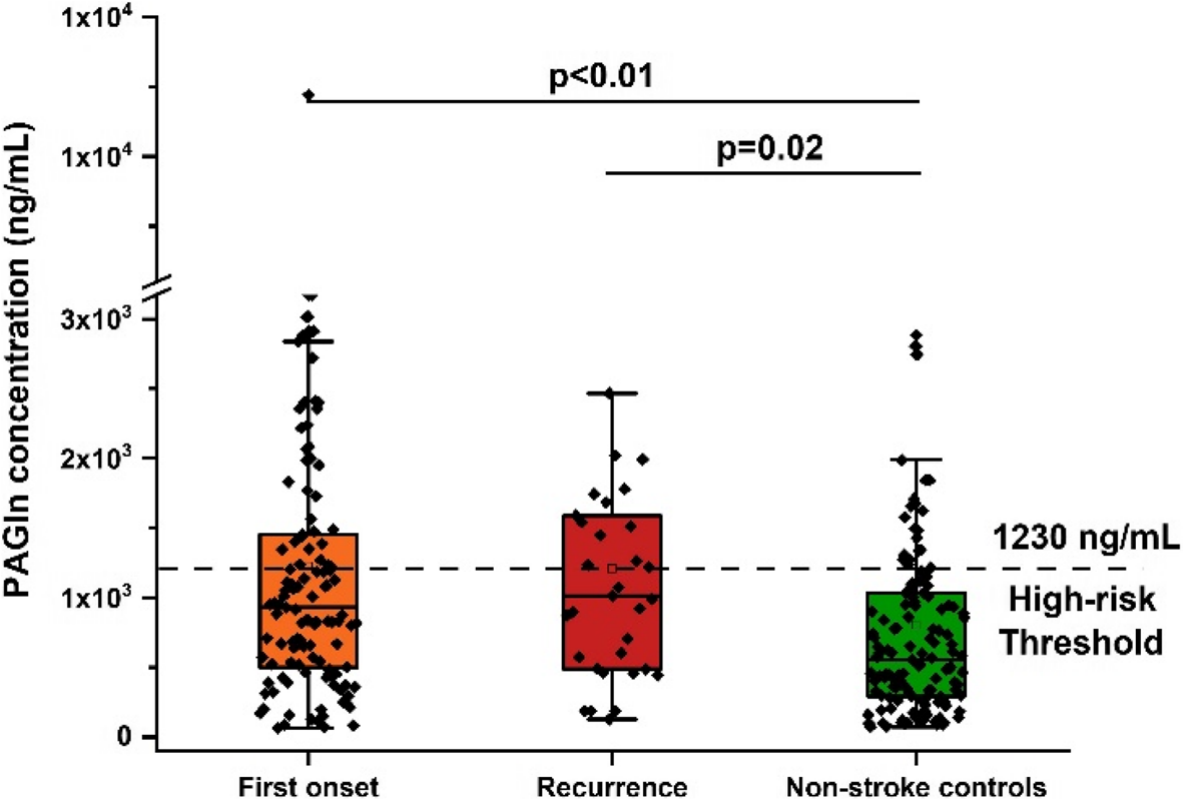
Comparison of plasma PAGln level among first-onset stroke subjects, recurrent stroke subjects, and non-stroke controls. The *p*-value for the comparison between every two groups was obtained by Student t-test. Error bars, transverse-line and quadrate in box plots indicate 1.5 IQR, median and mean, respectively

Compared to non-stroke controls, first-onset stroke patients and recurrent stroke patients are generally older and present higher blood pressure and PAGln levels (Table 1 and Fig. 1). Especially PAGln levels, the median of first-onset stroke patients and recurrent stroke patients was 933 ng/mL and 1014 ng/mL, almost twice that of the non-stroke controls (556 ng/mL). However, age, blood pressure, and blood glucose concentrations were not statistically different between first-onset and recurrent stroke patients.

### LASSO regression of PAGln level with baseline characteristics

**Fig. 2:**
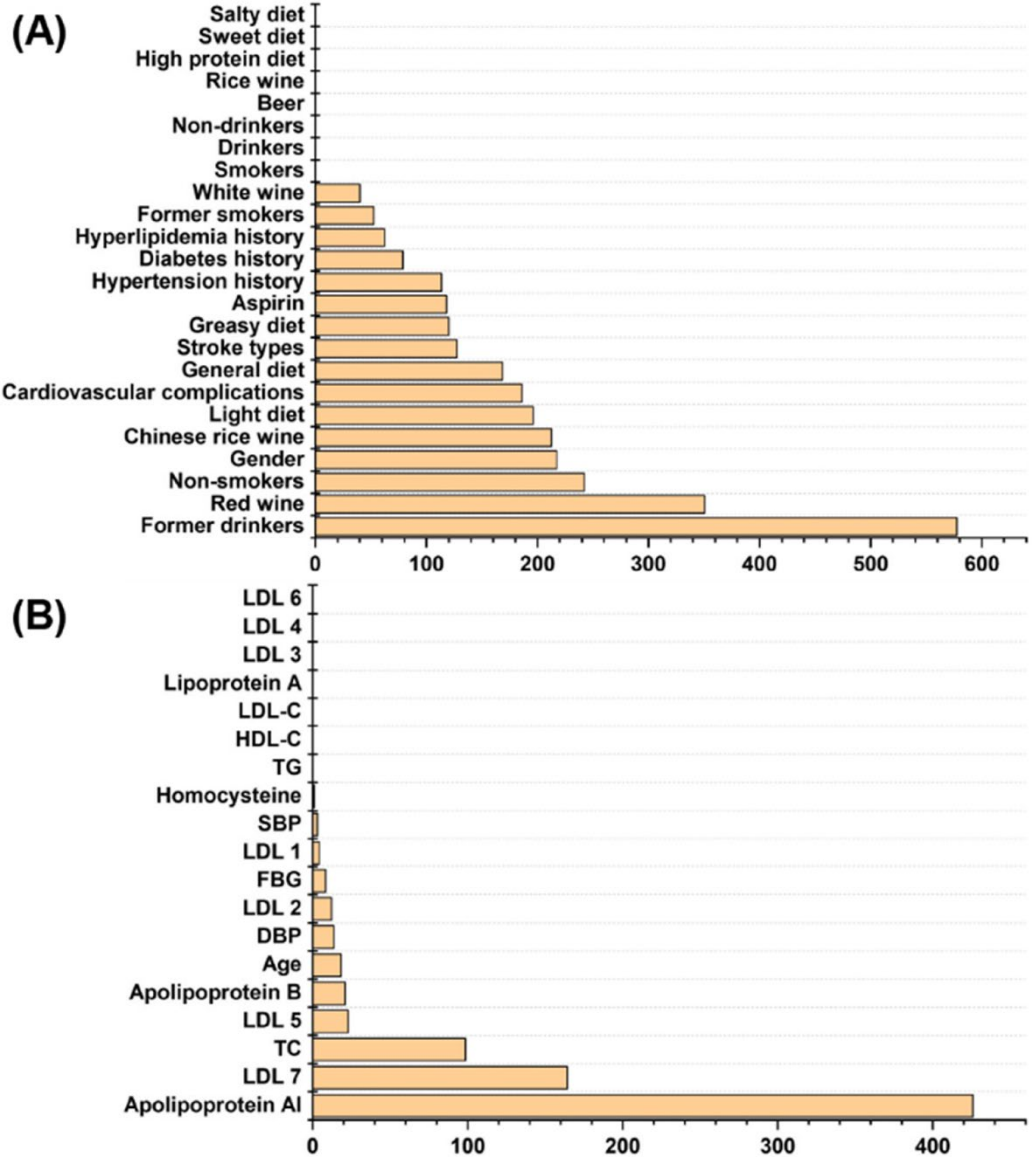
Lasso coefficients of (**A**) discrete variables and (**B**) continuous variables

**Fig. 3:**
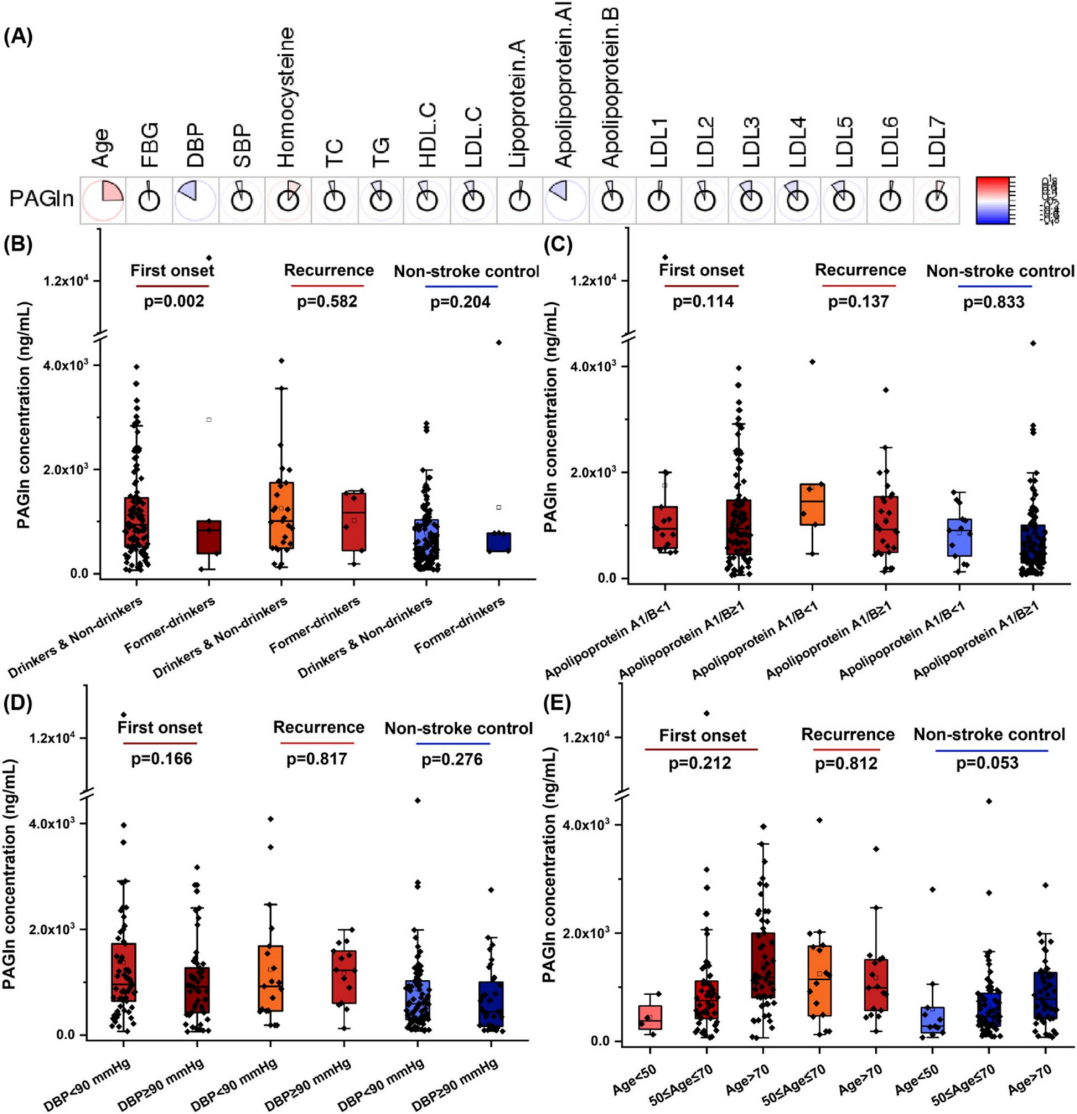
Comparison of plasma PAGln levels based on stroke risk factors among intragroup of first-onset stroke subjects, recurrent stroke subjects, and non-stroke controls. **A** Pearson correlation analysis between PAGln level and continuous variables of stroke risk factors. Intergroup comparison of plasma PAGln levels based on (**B**) drinking habits, **C** ratio of apolipoprotein A1/B, **D** DBP value, and **E** age. The *p* value for the comparison between every two groups was obtained by Student t-test, and among three groups was obtained by ANNOVA design. Error bars, transverse-line and quadrate in box plots indicate 1.5 IQR, median and mean, respectively

The regression analysis based on the least absolute shrinkage and selection operator (LASSO) were performed to demonstrate the potential contributions of baseline characteristics to the PAGln levels. Due to the potential imbalance of our baseline data, discrete variables and continuous variables were respectively performed LASSO regression with PAGln levels. As shown in Fig. 2, former-drinkers among discrete variables and apolipoprotein A1 among continuous variables showed the highest Lasso coefficient. For the correlation between continuous variables and PAGln levels, Person correlation analysis was also performed (Fig. 3A), and the results also proved that level of PAGln and apolipoprotein A1 have a significant correlation (q-value was − 0.162, the *p*-value was 0.04). In addition to apolipoprotein A1, Pearson correlation analysis also present that PAGln is related to subjects’ DBP and age. For DBP, the Pearson q-value was − 0.171 with a *p*-value of 0.04. For age variables, the Pearson q-value was − 0.248 with a *p*-value of 0.0005.

To further examine the effects of the above variables on PAGln levels, we stratified the cohort to assess changes in PAGln levels under these variables. Based on whether subjects are former drinkers, we found that the first-onset stroke patients had lower PAGln levels, with the *p*-value of 0.002 (Fig. 3B). However, this part of the subjects accounted for only 4.35% of first-onset stroke patients, indicating that there was a greater probability of accidental error. Although there is a significant correlation between the level of apolipoprotein A1 and PAGln, the ratio of apolipoprotein A1 and apolipoprotein B is usually used as a marker in clinical practice for the diagnosis of cardiovascular disease, with a threshold of 1.0. However, after subtyping based on the ratio of apolipoprotein A1/B, we found no significant differences in PAGln levels in the intra-group comparison of our cohort (Fig. 3C). Similarly, subtyping based on DBP, also no significant differences in PAGln levels can be found in the intra-group comparison (Fig. 3D). Different from the above variables, when subgrouping according to the age variable, we found that in the first-onset stroke group and the non-stroke group, PAGln levels tend to increase with age (Fig. 3E). However, we did not find a statistically significant difference via intra-group comparison based on the ANNOVA design, which may be due to the uneven distribution of subjects in different age groups, resulting in a decrease in the statistical power value.

### PAGln level was correlated with stroke severity

**Fig. 4:**
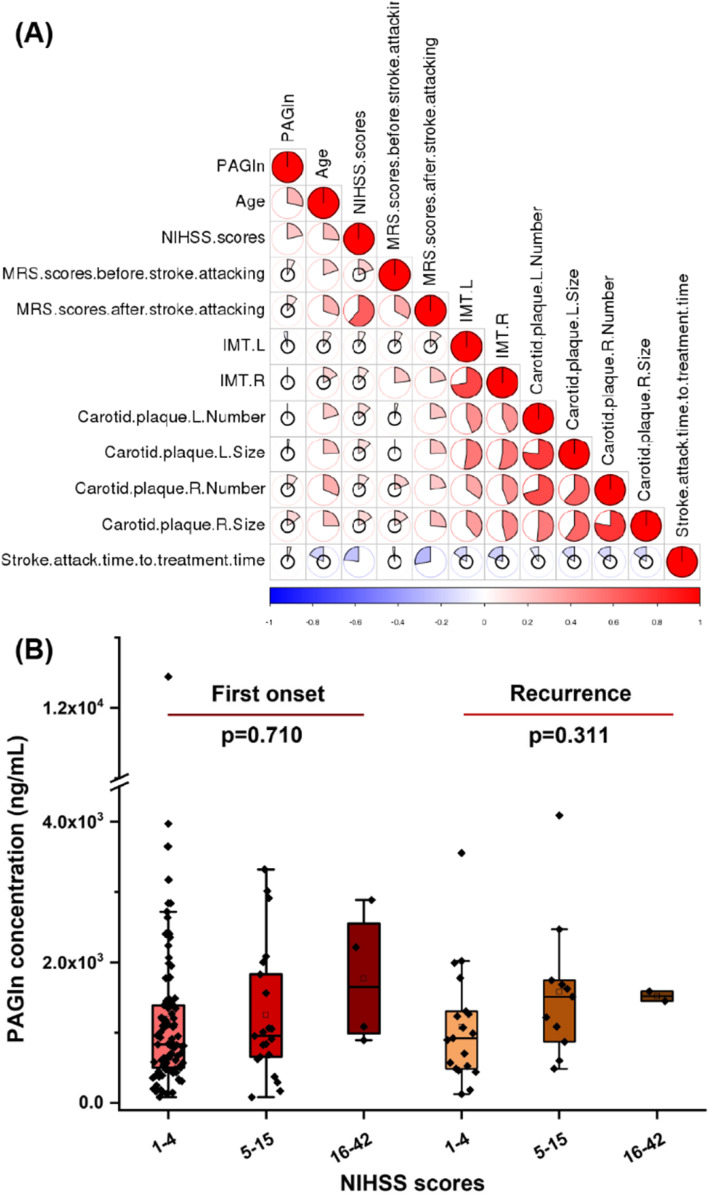
**A** Spearman correlation heatmap of plasma PAGln levels and stroke indicators. **B** Intergroup comparison of plasma PAGln levels based on NIHSS scores. The p value for the comparison among every three groups was obtained by ANOVA design (with a post hoc test). Error bars, transverse-line and quadrate in box plots indicate 1.5 IQR, median and mean, respectively

Next, we tried to demonstrate the relationship between plasma PAGln levels and stroke severity indicators, including NIHSS score, mRS score, intima-media thickness of the left and right carotid arteries, as well as the size and number of carotid plaques. The results of Spearman correlation analysis showed that PAGln levels were only significantly correlated with subjects’ age and NIHSS scores (Fig. 4A). Especially for NIHSS scores, which is a scale used to assess neurological impairment at the onset of stroke, has a q-value of 0.215 for the correlation with PAGln levels. In clinical practice, NIHSS scores 1–4 are considered mild strokes, 5–15 points are considered moderate strokes, and 16–42 points are considered severe strokes. Based on this standard, we subdivided the first-onset stroke patients and recurrent stroke patients into the 3 subgroups respectively, and then found that the median and mean of PAGln levels did increase with the severity of the stroke (Fig. 4B).

### PAGln level can be used to predict stroke recurrence

Based on the significant correlations between PAGln levels and stroke onset and/or stroke severity indicators, we next performed random forest machine learning, attempting to distinguish stroke patients from non-stroke controls. In addition to PAGln levels, traditional stroke risk factor indicators, including age, gender, living habits, complications of cardiovascular and cerebrovascular diseases, blood pressure and blood lipid parameters, etc., were also used in the machine learning process. In the process of machine learning, all variables are discretized according to whether that meet the health threshold, and then used for subject classification.

**Fig. 5:**
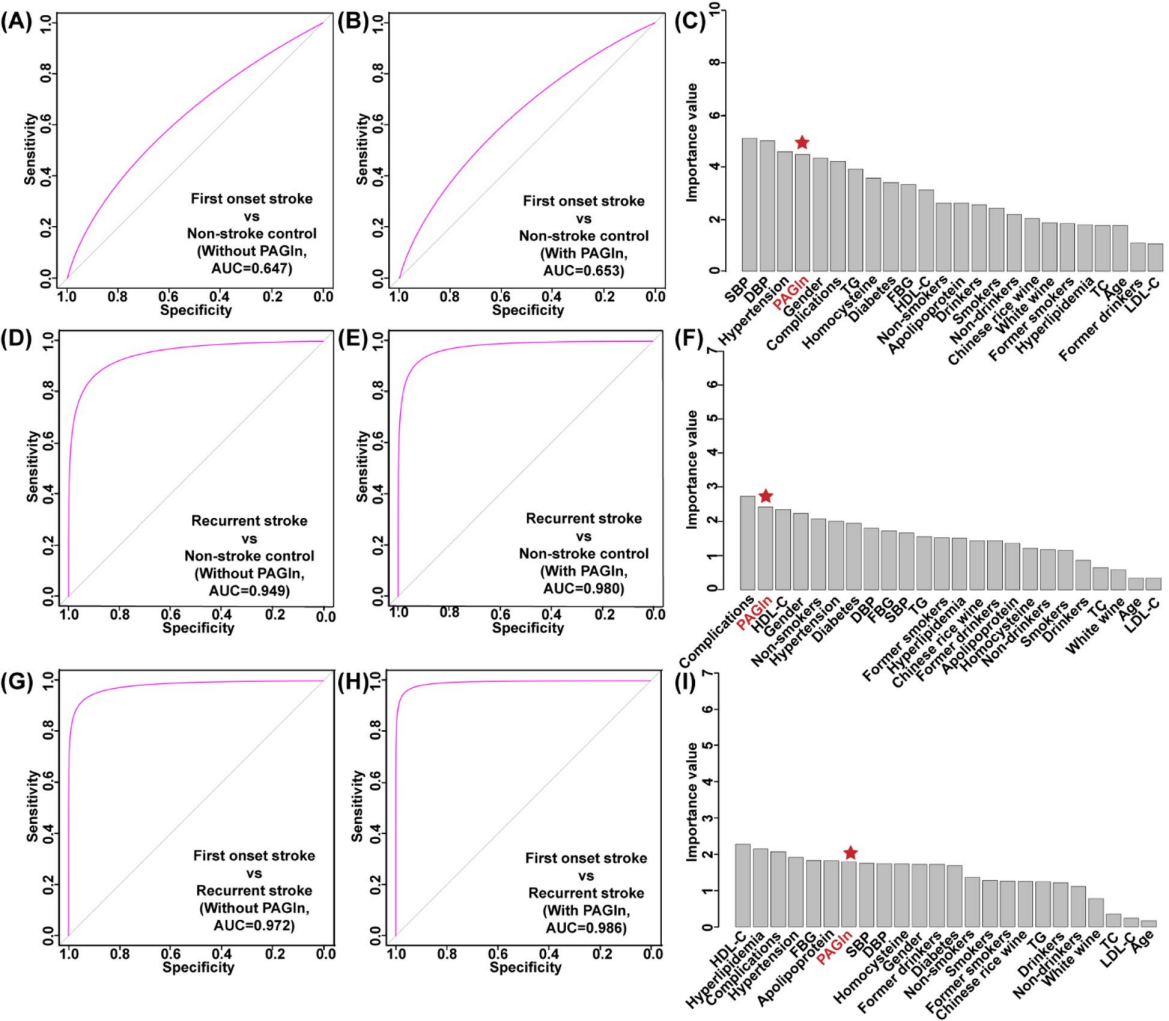
Random forest machine learning models were constructed to classify stroke-attack patients and non-stroke controls. Using the threshold of stroke risk indicators as learning parameters to distinguish (**A**) fist-onset stroke patients and non-stroke controls, **D** recurrent stroke patients and non-stroke controls, **G** fist-onset stroke patients and recurrent stroke patients. Using both of stroke risk indicators and PAGln level as learning parameters to distinguish (**B**) fist-onset stroke patients and non-stroke controls, **E** recurrent stroke patients and non-stroke controls, **H** fist-onset stroke patients and recurrent stroke patients. **C, F, I** Variable importance diagram

Regardless of whether the PAGln level is introduced, the machine learning model has poor discriminative performance for first-onset stroke patients and non-stroke controls, with an AUC value of only about 0.65 (Fig. 5A-C). However, for the comparison of recurrent stroke patients and non-stroke controls, machine learning model presented a better performance for classification. Before the introduction of PAGln level during machine learning, the AUC value of the classification model was 0.949 (Fig. 5D), and after the introduction of PAGln level, the AUC value was further improved to 0.980 (Fig. 5E). In this process, PAGln level was the second most important variable after cardiovascular and cerebrovascular complications (Fig. 5F). To account for the differences between the subjects of first-onset and recurrent stroke patients, we performed machine learning classification of the two groups of patients, and found the model had excellent classification performance, with AUC value of about 0.98 (Fig. 5G-H). The contribution of PAGln levels appears to be limited in the distinction between first-onset and recurrent stroke patients, and its importance is lower than that of lipid markers and complications of stroke (Fig. 5I). Despite the importance of stroke complications for stroke onset prediction, it is often difficult to use in dynamic monitoring applications. But, the PAGln level dynamic monitoring has significant advantages in this regard. Based on the previously reported PAGln risk threshold of 1230 ng/mL [8], we found that 42.4% of patients in the recurrent stroke cohort exceeded this value, whereas only 32.2% of the first-onset and 17.8% of non-stroke cohorts exceeded this threshold (Fig. 1).

## Discussion

Stroke is a cerebrovascular disease with high morbidity, high mortality and high disability rate worldwide. Its incidence prediction and prevention are important topics in the current medical field [1]. At present, primary prevention can be implemented for patients with ischemic stroke, including changes in lifestyle and diet, treatment of risk factors such as hypertension, diabetes, and lipid disorders, antiplatelet therapy for patients at high risk of cardiovascular and cerebrovascular disease [10]. For symptomatic ischemic stroke patients, secondary prevention can be implemented, including additional carotid artery surgery or stenting, closure of the patent foramen ovale after cryptogenic stroke, and medication for insulin resistance and intracranial stenosis [10]. In addition, because the metabolic mechanism of the onset of stroke has not been fully elucidated, imaging evidence is often used clinically to guide the prevention and treatment of stroke [6]. However, these methods have low temporal precision in the application of stroke onset monitoring, and the development of small molecule metabolites that can indicate stroke attacking can improve this situation. Among them, PAGln has recently been shown to have a well-defined stroke pathogenesis [8]. Further confirmation of the association between PAGln levels and stroke onset through population cohort studies has important clinical significance.

In this study, we established a LC-MS based targeted analysis method to detect the PAGln level in plasma samples, and further demonstrate the association between plasma PAGln levels and stroke attack (including first-onset and recurrence), using machine learning algorithm. Our results showed that PAGln were differentially enriched in stroke subjects, with the median level of 933 ng/mL in first-onset stroke patients and 1014 ng/mL in recurrent stroke patients, almost two times higher than non-stoke controls (median level of 556 ng/mL). Based on LASSO regression analysis and Pearson correlation analysis, we found that plasma PAGln level was an independent risk factor associated with stroke attack. Meanwhile, we found that a potential association of patients’ age and NIHSS score with plasma PAGln level. More importantly, as one of main contributing variable, PAGln levels can increase the precision of machine learning classification models in distinguishing recurrent stroke patients (but not first-episode stroke patients) from non-stroke controls.

Phenylalanine is the precursor of PAGln [11]. Most phenylalanine is absorbed in the small intestine, but the unabsorbed phenylalanine is first metabolized to phenylpyruvate by the intestinal microbiota in the large intestine, and then further metabolized to phenylacetic acid [8, 11]. After phenylacetic acid is absorbed by the portal venous system, it is metabolized in the liver to produce PAGln, which increases platelet reactivity and thrombosis through G protein-coupled receptors (including α2A, α2B, and β2-adrenergic receptors) [6, 8]. Recently, a cardiovascular disease cohort study (*n* = 5157) from the Cleveland Clinic revealed that plasma PAGln levels are related to cardiovascular disease and its adverse events [8]. A cohort study from the Chinese Han population (*n* = 595) also confirmed that plasma PAGln levels are related to white matter hyperintensity in patients with ischemic stroke [6]. In our study, we further demonstrated the association between PAGln levels in the Chinese Han population with recurrent strokes, and PAGln level differences relative to non-stroke controls.

At present, studies on the PAGln levels of stroke patients worldwide are very rare. Even though, our results are still within the threshold range given by Cleveland Clinic’s research (quartile interval was 1.82–4.92 μM, or about 455–1230 ng/mL) [8]. Compared with the previous Chinese stroke cohort [6], the PAGln levels of our cohort are relatively high than that reported (quartile interval was 1.21–3.34 μM, or about 303–835 ng/mL). The possible reason is that the subjects’ samples in our cohort were collected whithin 6 h after the stroke rescue, while the previous findings were the sampling within 14 days after the stroke attack. Moreover, with 1230 ng/mL as the high-risk threshold of cardiovascular disease adverse events [8], 32.2% of subjects in the first-onset stroke group and 42.4% of subjects in the recurrent stroke group met this standard, relative to 17.8% of non-stroke subjects meeting that.

For patients who have experienced a stroke, the greatest risk of death is stroke recurrence [12]. Therefore, screening people with high risk of stroke recurrence is very important for the prevention and rescue of stroke. Because of its low invasiveness, low cost, and great conveniences, the development of blood biomarkers has been widely studied for stroke-onset prediction in recent years, and a series of biomarkers including alanine transaminase [13], phospholipase A2 [14], and neutrophil to lymphocyte ratio [15], etc. have been reported in succession. However, with the in-depth research on intestinal microbiota, more and more evidence shows that the occurrence of cardiovascular adverse events, including but not limited to stroke, is closely related to the abnormal disturbance of intestinal microbiota and their metabolites [16]. The previously reported intestinal-microbes-associated cardiovascular disease markers, such as TMAO, often be interfered by a variety of external factors, such as diet [17–21], complications of cardiovascular diseases [22, 23], and therapeutic drugs [24–26]. However, in our cohort study, no statistical difference was found between PAGln level and the subject’s living habits, eating preferences, concomitant diseases (hypertension, hyperlipidemia, and diabetes), TOAST subtype, and aspirin treatment (Table 1, Figs. S1 and S2). Therefore, daily monitoring of plasma PAGln levels in patients who have experienced a stroke may be as important as monitoring blood glucose in diabetic patients in the future.

In fact, we indeed found that subjects’ age and NIHSS score seemed to be related to plasma PAGln levels. As we all know, the age factor is an important factor in the incidence of stroke [27]. The incidence of stroke in the 50–70-year-old Chinese population is indeed higher than that of the population under the age of 50. NIHSS scores is a scale used to assess neurological impairment at the onset of stroke, its correlation with the level of PAGln can partly explain the indication effect of PAGln level on stroke attacking. Therefore, it is still necessary to further construct age grouping and NIHSS grouping based on power analysis in the follow-up research, to illustrate the influence of these two covariates on PAGln levels. Especially the relationship between PAGln level and NIHSS score, further confirmation of this relationship will have important guiding significance for primary and secondary prevention of stroke.

This study has several limitations. First, this is a single-center cross-sectional study. Large-scale multi-center parallel verification and long-term follow-up will further enhance the level of evidence for the conclusions of this study. Second, the proportion of young patients is relatively small compared to that of middle-aged and elderly patients. The number of subjects needs to be further expanded to better balance the effects of age on the results. In addition, refined diet management and comprehensive intestinal microbiota information should be implemented to minimize the “background value” introduced by non-disease factors.

## Conclusions

Ischemic stroke is the typical stroke subtype in the China Han population, which also is one of the main lethal and disabling diseases. Recently, PAGln has been confirmed to be related to the occurrence of cardiovascular diseases and their adverse events, but there is a lack of Chinese cohort evidence. Our study found that stroke-attacking patients with first onset and recurrence have higher PAGln levels than non-stroke controls. The level of PAGln has negligible associations with the subjects’ living habits, dietary preferences, complications of cardiovascular disease, and aspirin treatment history. The subject’s age and NIHSS score may affect their plasma PAGln levels. By combining PAGln levels with stroke risk factors, it is possible to more accurately distinguish patients with recurrent stroke from non-stroke controls. Therefore, PAGln may be a potential monitoring indicator of stroke attack, and also may have guiding significance for the prevention and treatment of stroke.

## Supplementary Information

**Additional file 1: Table S1.** Baseline characteristics of the cohort.

**Additional file 2: Fig. S1.** Intergroup comparison of plasma PAGln levels in first stroke group (A) and recurrent stroke group (B) based on TOAST subtype. **Fig. S2.** Intergroup comparison of plasma PAGln levels in first stroke group and recurrent stroke group according to aspirin use.

## Data Availability

The detail baseline information of our cohort is available in the Supplementary information 1. The subtype analysis results about TOAST criteria or aspirin use is available in Supplementary information 2.

## Abbreviations

CVD: Cardiovascular diseases
PAGln: Phenylacetyl glutamine
HDL-C: High-density lipoprotein-cholesterol
LDL-C: Low-density lipoprotein-cholesterol
IMT: Intima-media thickness
TMAO: Trimethylamine N-oxide
FBG: Fasting blood-glucose
DBP: Diastolic blood pressure
SBP: Systolic blood pressure
TC: Total cholesterol; TG: triacyl-glycerol
