# Supplementary material for: Phenylacetyl glutamine: a novel biomarker for stroke recurrence warning
Source: BMC Neurol. 2023 Feb 16;23:74. doi: 10.1186/s12883-023-03118-5 (PMC9933389; doi:10.1186/s12883-023-03118-5)
Supplement: Supplementary file 2 — Additional file 2: Fig. S1. Intergroup comparison of plasma PAGln levels in first stroke group (A) and recurrent stroke group (B) based on TOAST subtype. Fig. S2. Intergroup comparison of plasma PAGln levels in first stroke group and recurrent stroke group according to aspirin use. [file 12883_2023_3118_MOESM2_ESM.pdf]

Supporting Information 2

Phenylacetyl Glutamine: A Novel Biomarker for Stroke Recurrence Warning

Li Ma,[1*] Guoping Fu,[1] Rongrong Liu,[1] Feng Zhou,[1] Shiye Dong,[2,3] Yang Zhou,[1] Jingwei Lou,[2*] Xinjun Wang, [2, 4, 5*]

1 Department of Neurology, Shaoxing Second Hospital, The Second Affiliated Hospital of Shaoxing University of Arts and Sciences, Shaoxing 312000, China.

2 Shanghai Zhangjiang Institute of Medical Innovation, Shanghai 201204, China.

3 Molecular Medicine Center, Shaoxing Second Hospital, The Second Affiliated Hospital of Shaoxing University of Arts and Sciences, Shaoxing 312000, China.

4 Translational Medical Center for Stem Cell Therapy and Institute for Regenerative Medicine, Shanghai East Hospital, Shanghai Key Laboratory of Signaling and Disease Research, School of Life Sciences and Technology, Tongji University, Shanghai 200092, China.

5 Shanghai Institution of Gut Microbiota Research and Engineering Development, Tenth People's Hospital of Tongji University, Tongji University School of Medicine, Shanghai 200072, China.

* Corresponding to Xinjun Wang (xjwang16@ fudan.edu.cn), Jingwei Lou (jingweilou@biotecan.com) and Li Ma (malijinchi@sina.com).

Contents

S1. Effect of TOAST stroke subtypes on PAGln levels…………………………………………………………………..... 2

S2. Effect of aspirin use on PAGln levels……………………………………………………………………...……….... 3

S1. Effect of TOAST stroke subtypes on PAGln levels.

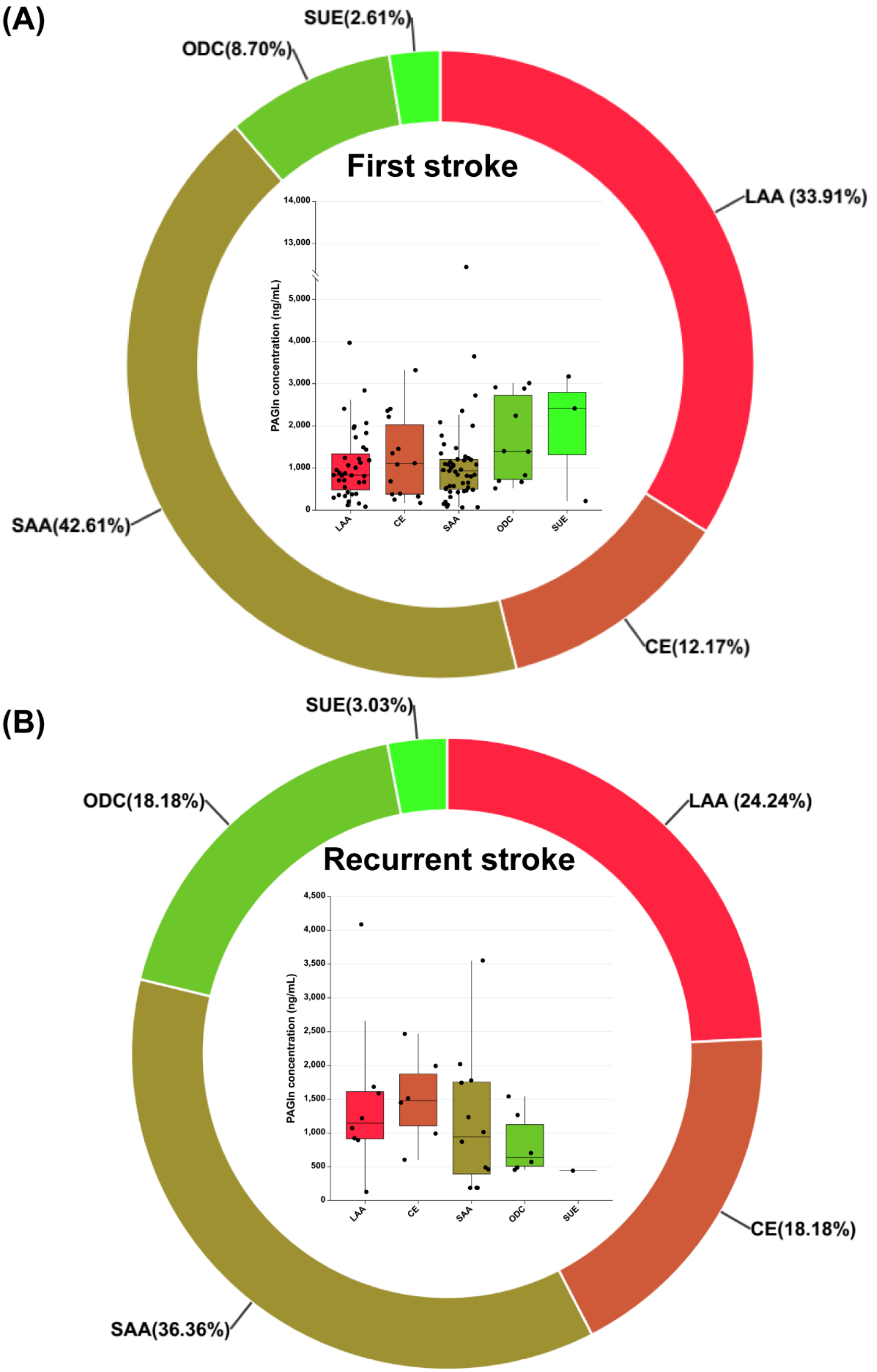

Figure S1. Intergroup comparison of plasma PAGln levels in first stroke group (A) and recurrent stroke group (B) based on TOAST subtype.

S2. Effect of aspirin use on PAGln levels.

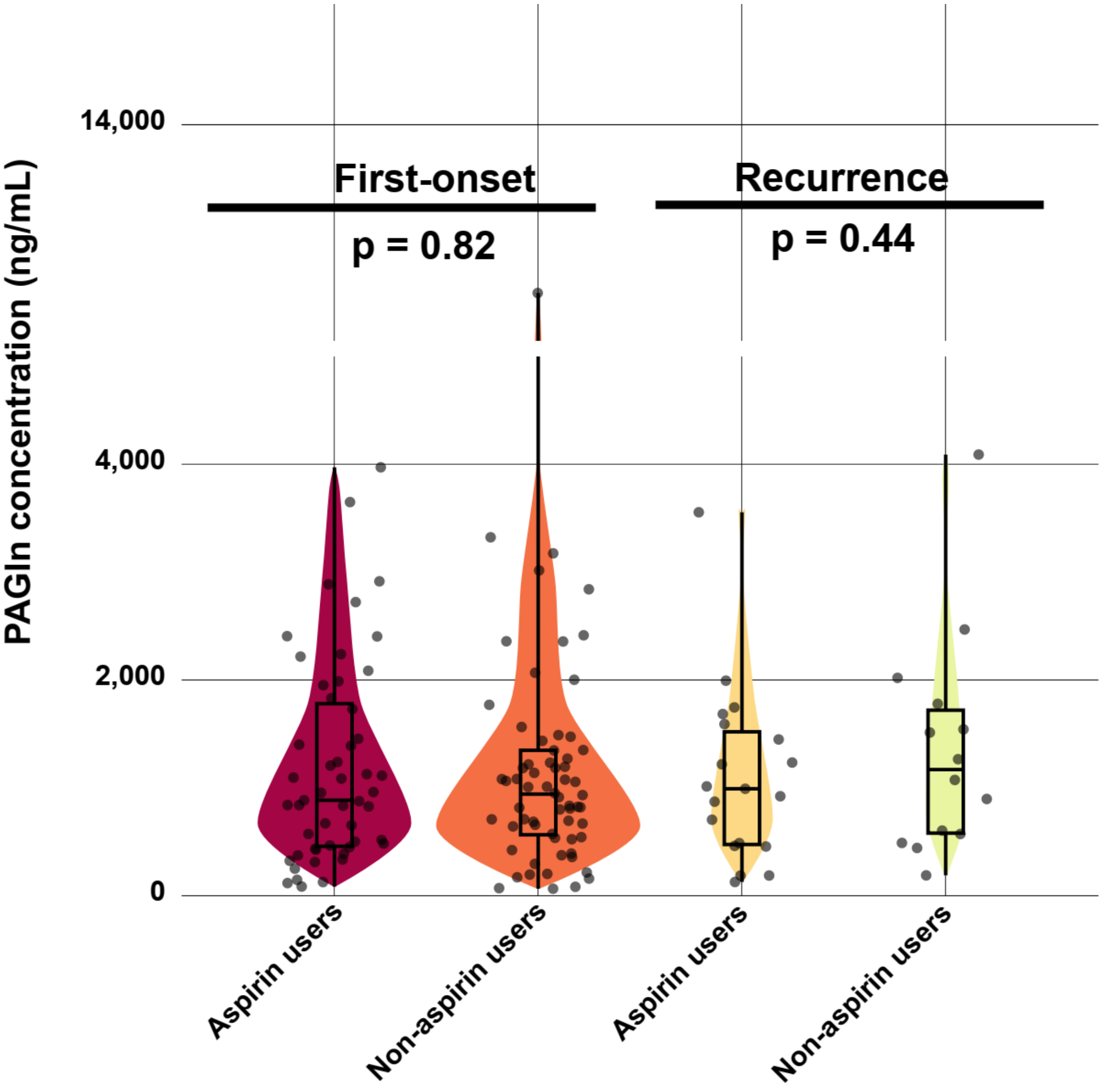

Figure S2. Intergroup comparison of plasma PAGln levels in first stroke group and recurrent stroke group according to aspirin use.
